# Is Health Information Exchange Use by Hospitals and Home Health Agencies Associated with Lower Readmission Rates?

**DOI:** 10.1093/geroni/igaa057.3360

**Published:** 2020-12-16

**Authors:** Christine Jones, Jacob Thomas, Marisa Roczen, Kate Ytell, Mark Gritz

**Affiliations:** 1 Rocky Mountain Regional VA Medical Center, Aurora, Colorado, United States; 2 University of Colorado, Aurora, Colorado, United States

## Abstract

For older adults transitioning from the hospital to home health agencies (HHAs), clinical information exchange is key for optimal transitional care. Hospital and HHA participation in regional health information exchanges (HIEs) could address fragmented communication and improve patient outcomes. We examined differences in characteristics and outcomes for patients with either Medicare or Medicare Advantage (MA) insurance who transitioned from hospitals to HHAs based on HIE participation with 2014-2018 data from the Colorado All Payer Claims Database. We performed analyses including chi square and t tests to compare patient characteristics and 30-day readmission rates for high versus lower HIE use, determined by HIE participation (+) and non-participation (-) among HHAs and hospitals: High HIE use dyads (Hospital+/HHA+) were compared to lower HIE use dyads (Hospital+/HHA-, Hospital-/HHA+, Hospital-/HHA-). We identified 57,998 care transitions from 123 acute care hospitals to 71 HHAs. On average, patients were 75 years old, had a three day hospital length of stay, over half were female (58%), 82% had Medicare and 18% had MA insurance. Although most characteristics were similar between high versus lower HIE use dyads, high HIE use dyads had a higher proportion of Medicare patients compared to the lower HIE use dyads (85% vs 79%, p <0.001). Thirty-day readmissions were 12.4% for care transitions that occurred among high HIE use dyads (n=27,784) compared to 12.8% among lower HIE use dyads (n=32,929, p=0.102). For adults transitioning from hospitals to HHAs among high HIE use dyads, a trend toward lower 30-day readmission rates was identified.

